# Is Intragastric Botulinum Toxin A Injection Effective in Obesity Treatment?

**DOI:** 10.1155/2020/2419491

**Published:** 2020-10-01

**Authors:** Bulent Kaya, Nuriye Esen Bulut, Mahir Fersahoglu

**Affiliations:** ^1^Hisar Intercontinental Hospital, Department of General Surgery, Istanbul, Turkey; ^2^Fatih Sultan Mehmet Training and Research Hospital, Department of General Surgery, Istanbul, Turkey

## Abstract

**Aims:**

The objective of this prospective study was to evaluate the efficacy of intragastric botulinum toxin A (BTX-A) injection for the treatment of obesity.

**Materials and Methods:**

The study was performed between January and August 2019. This is a prospective study. After 6–12 hours of fasting, the patients were submitted to upper GI endoscopy under sedation for the injection of BTX-A. A total of 250 U of BTA-X was diluted with 10 ml of 0.9% saline. Injections were administered into the gastric antrum, each containing 1 ml of prepared solution (25 U BX-A + 1 ml saline). Continuous data were compared using a two-sample *t*-test. Statistical significance was determined as *P* ≤ 0.05. All statistical analysis was performed using SPSS for Windows 22.1 software (SPSS, Chicago, IL, USA).

**Results:**

A total of 56 patients were studied. Mean weight before gastric Botox was 85.25 ± 14.02, and mean weight after gastric Botox was 76.98 ± 12.68. Mean weight loss was approximately 9 kg in studied patients. BMI decreased about 3 units. The mean time for maximum weight loss was 60.39 ± 37.43 days. A total of 49 patients (87.5%) had reported decrease in appetite and early satiety. About 53.6% of patients were satisfied. No complications resulting from the endoscopic procedure were observed in this series.

**Conclusions:**

Intragastric BTX-A injection can be beneficial in weight loss. It is a minimally invasive, cost-effective procedure, without serious side effects.

## 1. Introduction

Obesity has become an epidemic disease in the past years. Genetic, social, psychological, and behavioural factors are important in obesity. It is well known that obesity increases the risk of some disorders including diabetes, arterial hypertension, hyperlipidemia, sleep apnea syndrome, and cerebral stroke [1]. There is no gold standard pharmacological treatment for obesity up to date.

Botulinum neurotoxin, produced by the Gram-positive, anaerobic bacterium *Clostridium botulinum*, has been used widely for many diseases. It was first injected into extraocular muscles for treatment of ocular strabismus [2]. It mainly inhibits the muscular contractions of smooth and striated muscles by decreasing acetylcholine release at the neuromuscular junction. It was also shown that botulinum toxin is effective in the treatment of some diseases with hyperactivity of the gastrointestinal smooth muscles such as achalasia and chronic anal fissure [3–5].

After the use of botulinum toxin A (BTX-A) in experimentally obese rat models, endoscopic intragastric BTX-A injection has been attempted for the treatment of morbid obesity [6, 7]. The mechanism of action is related to gastric emptying delay and early satiety, resulting in weight loss. There have been few conflicting results about intragastric Botox injection including body weight reduction, gastric emptying, induction of early satiety, and weight gain after the procedure. The objective of this prospective study was to evaluate the efficacy of intragastric BTX-A injection for the treatment of obesity.

## 2. Materials and Method

This study was performed between January and August 2019. All patients gave informed consent according to ethical committee principles. Inclusion criteria included age between 18 and 65 years and body mass index (BMI) ˃ 25 kg/m^2^. Patients who were pregnant and lactating and had myopathy or neuromuscular disorder, hypersensitivity to BTX-A, cardiovascular disease, and peptic ulcer disease detected in endoscopy were excluded. Body weight and height were measured, and BMI was found out before the procedure (body weight (kg)/height (m^2^)). Body weight and BMI were measured monthly until the end of the study (5 months).

### 2.1. Procedure

After 6–12 hours of fasting, the patients were submitted to upper GI endoscopy under sedation for the injection of BTX-A. A total of 250 units of BTX-A was diluted with 10 ml of 0.9% saline. Injections were administered into the gastric antrum, each containing 1 ml of prepared solution (25 U BTX-A + 1 ml saline). Technically, the antral region of the stomach was punctured, and BTX-A was injected into the gastric wall slowly, using a standard 5 mm sclerotherapy needle in a circular direction. Usually, 8–10 injections were given with routine three punctures in the posterior gastric wall, three punctures in the anterior gastric wall, two punctures in the lesser curvature, and two punctures in the greater curvature.

There were no significant acute complications during the procedures except a minor bleeding that stopped without any intervention. All patients were observed for 4 h after the procedure. Only one patient complaining of abdominal pain and vomiting was observed for 12 h. All patients were suggested to visit a dietitian immediately after the endoscopic procedure.

### 2.2. Diet and Follow-Up

The patients were suggested to take the pure liquid diet within 72 hours after the procedure. All patients were consulted by the dietitian. At the end of 3 days, a 1200 kcal (protein 30%, carbohydrate 30%, and lipid 40%) was given. The patients were offered to follow the diet for 6–9 months and controlled by the dietitian every 3 weeks.

All patients were invited for control at the end of the first and fifth months.

### 2.3. Statistical Analysis

We performed descriptive analyses of all of the study variables. Quantitative variables are expressed as means, with minimum and maximum values, or as means with standard deviations as appropriate. Continuous data were compared using Student's *t*-test. Statistical significance was determined as *P* ≤ 0.05. All statistical analysis was performed using SPSS for Windows 22.1 software (SPSS, Chicago, IL, USA).

## 3. Results

**Figure 1:**
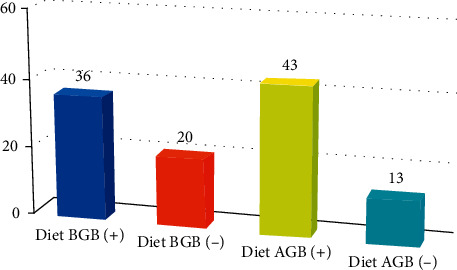
Dietary practice before gastric Botox (BGB) and after gastric Botox (AGB).

**Figure 2:**
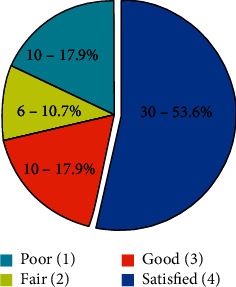
Patient satisfaction scores and percentages.

A total of 56 patients were studied. There were 50 (89.3%) females and 6 (10.7%) males. The mean age was 38.97 ± 9.90 (22–61 years). The demographic findings of all patients are shown in Table 1. Mean weight before intragastric Botox was 85.25 ± 14.02, and mean weight after intragastric Botox was 76.98 ± 12.68. Mean weight loss was approximately 9 kg in studied patients. BMI decreased about 3 units. The mean time for maximum weight loss was 60.39 ± 37.43 days (Table 2). Appetite and early satiety were questioned. A total of 49 patients (87.5%) had reported a decrease in appetite and early satiety (Table 3). Dietary practices are shown in Figure 1. The patient satisfaction was measured with scoring (4: satisfied; 3: good; 2: fair; 1: poor). About 53.6% of patients were satisfied (Figure 2). No complications resulting from the endoscopic procedure were observed in this series.

## 4. Discussion

BTX-A has already been used for decades in both esthetic and therapeutic procedures [8–11]. The neuromuscular blockade caused by BTX-A provokes the thesis that this agent can be applied to the gastric wall, resulting in the feeling of bloating, delayed gastric emptying, early satiety, and weight loss. The main advantage of using the intragastric injection of BTX-A would be the absence of serious adverse events related to the procedure [12, 13].

Previous studies evaluating intragastric injection of BTX-A as a treatment for obesity have been associated with conflicting results. Rollnik et al. published the first report of the use of BTX-A for the treatment of obesity [14]. Total body weight loss was 8.9% (9 kg) of the initial body weight. In an experimental study, Gui et al. showed that the intraparietal administration of BTX-A into the gastric antrum causes a significant reduction of food intake and decrease of body weight [6]. Albani et al. applied 500 IU of BTX-A to 8 patients in a small series and found a total weight loss of approximately 4 kg at 1-month follow-up [15]. On the other hand, there are studies showing that intragastric Botox treatment is ineffective in weight loss. Bustamante et al. designed a meta-analysis with four randomized clinical trials [12]. They concluded that homogeneous randomized controlled studies did not find superiority of BTX-A versus placebo [6, 16, 17]. de Moura et al. also studied the effect of BTX-A in super-obese patients. They concluded that intragastric injection of BTX-A does not seem to be an effective method in achieving preoperative weight loss in these patients [18].

As far as we know, our study is one of the largest series published in the literature about the intragastric Botox injection. A total of 56 patients were studied prospectively. If we list the important results of the study, the weight loss after procedure was found to be approximately 9 kg. This finding is similar to the study results of Rollnik et al. This weight loss amount may suggest that intragastric Botox application will be more suitable especially for class 1 patients.

Diet is one of the important issues in the treatment of obesity. In our study, a low-calorie diet was recommended to all patients after the procedure. One of the interesting results of the study was that there was no statistical difference in terms of weight loss in patients who were on a diet or not (*P* > 0.05).

One of the most curious topics in this study was the effect of Botox on appetite. After the intragastric Botox procedure, 47 of 56 patients stated that their appetite decreased. The importance of decreased appetite in weight loss is well known. After sleeve gastrectomy, which is an important bariatric surgery method, ghrelin hormone level and appetite decrease. Gastric antral contractility is an important component of normal gastric motility. Reduced antral motility appears to prolong gastric emptying. Intragastric BTX-A injection inhibits contractility of the gastric muscle. So we also injected BTX-A into the antral region of the stomach.

More than half of the patients reported a high satisfaction score after the intragastric Botox procedure. It is also important that no serious complications are seen during or after procedures.

In this study, it was observed that intragastric Botox administration with diet showed a clear effect on appetite and provided an average weight loss of 9 kg. But there have been patients in the series who had no weight loss and no decreased appetite. Diet can also be a confusing factor when investigating the effectiveness of Botox in weight loss. These results indicate that larger randomized prospective studies are needed to demonstrate the effectiveness of intragastric Botox.

In conclusion, it is shown that the intragastric BTX-A injection can be beneficial in weight loss. It is a minimally invasive, cost-effective procedure, without serious side effects. This study has some limitations.

## Figures and Tables

**Table 1:** Demographic findings of patients.

Finding	Mean ± SD	Number
Age	38.97 ± 9.90	56
Gender		6 male/50 female (10.7%–89.3%)
Obesity in family		19 patients (33.9%)
Psychiatric problems		5 patients (8.9%)
Comorbidities		
Diabetes mellitus		6 patients (10.7%)
Hypertension		4 patients (7.1%)
Hypothyroid		2 patients (3.5%)
Polycystic ovary syndrome		1 patient (1.7%)

**Table 2:** Average weight and BMI loss.

	Mean ± SD	Min–max
Weight before gastric Botox	85.25 ± 14.02	61–123 kg
Weight after gastric Botox	76.98 ± 12.68	54–123 kg
Average time for weight loss	60.39 ± 37.43	7–150 days
BMI before gastric Botox	30.79 ± 4.25	25–43.6
BMI after gastric Botox	27.95 ± 3.76	38.3–20.6

**Table 3:** Effect of gastric Botox on appetite of patients.

	Number of patients	Appetite
Appetite before gastric Botox	33	High
	13	Normal

Appetite after gastric Botox	49	Decreased
	7	Not changed

## Data Availability

All prospective data used to support the findings of this study are included within the article and are available from the corresponding author upon request.
